# Effect of the Interaction between Viral PB2 and Host SphK1 on H9N2 AIV Replication in Mammals

**DOI:** 10.3390/v14071585

**Published:** 2022-07-21

**Authors:** Yong Zhou, Weihua Gao, Yan Sun, Yuxin Guo, Yuping Wu, Juan Pu

**Affiliations:** 1 Key Laboratory for Prevention and Control of Avian Influenza and Other Major Poultry Diseases, Ministry of Agriculture and Rural Affairs, College of Veterinary Medicine, China Agricultural University, Beijing 100193, China; zhouyong@cau.edu.cn (Y.Z.); gwh212@163.com (W.G.); yyyyyan111@163.com (Y.S.); yuuuxin_guo@163.com (Y.G.); 2 College of Life Science and Basic Medicine/Center for Biotechnology Research, Xinxiang University, Xinxiang 453003, China

**Keywords:** H9N2 avian influenza virus, DK1-like lineage PB2 gene, transcriptomics, SphK1

## Abstract

The H9N2 avian influenza virus (AIV) is currently widespread worldwide, posing a severe threat to the poultry industry and public health. Reassortment is an important way for influenza viruses to adapt to a new host. In 2007, the PB2 gene of H9N2 AIV in China was reassorted, and the DK1-like lineage replaced the F/98-like lineage, forming a dominant genotype of G57. This genotype and its reassortants (such as H7N9, H10N8 and H5N6) showed higher mammalian adaptation, and caused increased human infections. However, the adaptive mechanisms of the DK1-like lineage PB2 gene remain unclear. Here, we confirmed that the PB2 lineage of the H9N2 AIV currently prevalent in China still belongs to the DK1-like lineage and, compared with the previously predominant F/98-like lineage, the DK1-like lineage PB2 gene significantly enhances H9N2 AIV to mammalian adaptation. Through transcriptomic analysis and qRT–PCR and western blot experiments, we identified a host factor, sphingosine kinase 1 (SphK1), that is closely related to viral replication. SphK1 inhibits the replication of DK1-like PB2 gene H9N2 AIV, but the ability of SphK1 protein to bind DK1-like PB2 protein is weaker than that of F/98-like PB2 protein, which may contribute to H9N2 AIV containing the DK1-like PB2 gene to escape the inhibitory effect of host factor SphK1 for efficient infection. This study broadens our understanding of the adaptive evolution of H9N2 AIV and highlights the necessity to pay close attention to the AIV that contains the adaptive PB2 protein in animals and humans.

## 1. Introduction

The H9N2 avian influenza virus (AIV) has become globally widespread, not only causing severe losses to the poultry industry, but also posing a serious threat to public health security [1,2,3]. During the two decades with the epidemic in China, H9N2 AIV have undergone complex genetic reassortments and mutations, resulting in multiple genotypes [4,5,6,7]. Notably, the pathogenicity of the H9N2 AIV isolated from avian flocks in China to ferrets increased significantly during 2009–2013 [8]. Consistent with this, the cases of human infection with H9N2 AIV sharply increased from 2009 to 2021 [1,9]. Moreover, H9N2 AIV provided internal genes for novel H7N9, H10N8 and H5N6 avian influenza viruses that emerged in China, and which can directly infect humans, in 2013 and 2014 [10,11,12]. The mammalian adaptation of the H9N2 virus is closely related to the PB2 gene [13,14]. Our previous studies have shown that the PB2 gene of H9N2 AIV in China was reassorted in 2007, and the DK1-like PB2 lineage replaced the F/98-like PB2 lineage, subsequently forming a dominant genotype of G57 [5]. Liu et al. found that the DK1-like PB2 gene promotes the nuclear transport efficiency of the H9N2 virus [15], but the interaction and connection between the DK1-like PB2 protein and host factors have not been reported.

The interaction between viral proteins and host factors is a process of virus adaptation, and the strength of this interaction can significantly affect influenza virus replication in cells. According to the different effects on influenza virus replication, these host factors can be roughly divided into the following categories: one is a host factor that assists virus replication, and the other is a host immune response-related factor that inhibits virus replication. The PB2 protein is considered to be the main determinant of influenza virus virulence and mammalian fitness [16], and it is involved in the interaction with a variety of host factors in the process of viral functioning. The growth and replication of influenza virus must depend on host cells. At the same time, there are also a series of host factors in the body that are resistance to virus invasion and replication. Revealing the role of the host factors related to viral infection will provide guidance for the prevention and control of the influenza virus. The influenza virus activates related cell signaling pathways during its interaction with host cells, thereby inducing changes in host cell lipids [17,18,19]. Through transcriptomic analysis and experimental verification, we screened the sphingosine kinase 1 (*SphK1*) gene. The SphK1 protein encoded by the *SphK1* gene is a lipid kinase that regulates the conversion of Sph to S1P. The SphK1/S1P axis has well-described roles in cell signaling, the cell death/survival decision, the production of a proinflammatory response, immunomodulation and control of vascular integrity; it also regulates signaling pathways, such as Raf/MEK/ERK, NF-κB and PI3K/AKT/mTOR [20,21,22]. Therefore, studying the changes and roles of host SphK1 during infection with different lineages of the PB2 gene H9N2 AIV is beneficial for revealing the reasons for the enhanced mammalian adaptation of the DK1-like PB2 gene H9N2 AIV and providing new insights for understanding the host’s antiviral mechanism.

## 2. Materials and Methods

### 2.1. Sequence Collection and Alignment

All PB2 gene sequences of H9N2 AIV isolated in China (1994–2020) were obtained from the Global Initiative on Sharing Avian Influenza Data (www.gisaid.org (accessed on 28 June 2021) and the Influenza Virus Resource at the National Center for Biotechnology Information (NCBI) (www.ncbi.nlm.nih.gov/genomes/FLU (accessed on 28 June 2021) (Table S1) and were used for further analysis. All duplicated submissions were removed by identifying sets of isolates with identical viral names. The resulting sequences were aligned using MUSCLE v3.7 [23], manually adjusted to correct frame shift errors and subsequently translated. Downstream phylogenetic analyses were performed on coding regions of the alignments containing few gaps across sequences.

### 2.2. Phylogenetic Analysis and Clade Classification

IQTREE version 1.6 was used to construct the maximum likelihood phylogenetic trees, applying the best-fit general time-reversible model of nucleotide substitution with gamma-distributed rate variation among sites (GTR + I + G) and performing ultrafast bootstrap resampling analysis (1000 replications) [24]. Phylogenetic trees were visualized and annotated by using FigTree version 1.4.4, Adobe Illustrator 2021 and Interactive Tree Of Life (iTOL) [25].

### 2.3. Plasmids and Cells

Protein expression plasmids SphK1-HA-pRK5, SphK1-pRK5 (SphK1-Flag-pRK5), F/98-PB2-Flag-pRK5 and DK1-PB2-Flag-pRK5 were generated by subcloning the corresponding coding segment into the HA-pRK5 or Flag-pRK5 empty vector. Human embryonic kidney (293T) cells, human lung adenocarcinoma epithelial (A549) cells, Madin-Darby Canine Kidney (MDCK) cells and chicken embryo fibroblast (DF-1) cells were maintained in Dulbecco’s modified Eagle’s medium (DMEM; Gibco, Carlsbad, CA, USA) supplemented with 10% fetal bovine serum (FBS; Gibco, Carlsbad, CA, USA), 100 U/mL of penicillin and 100 μg/mL of streptomycin at 37 °C in a 5% CO_2_ atmosphere.

### 2.4. Polymerase Activity Assay

A dual-luciferase reporter assay system (Promega, Madison, WI, USA) was used to compare the polymerase activities of different viral RNP complexes [26]. PB2, PB1, PA and NP gene segments of indicated viruses were separately cloned into the pCDNA3.1 expression plasmid. PB2, PB1, PA and NP plasmids (125 ng each plasmid), along with the pLuci luciferase reporter plasmid (10 ng) and the renilla internal control plasmid (2.5 ng), were used to transfect 293T cells or DF-1 cells. 293T cell cultures were incubated at 33 °C or 37 °C; DF-1 cell cultures were incubated at 37 °C or 39 °C. Cell lysates were analyzed 24 h post-transfection for firefly and renilla luciferase activities using GloMax 96 microplate luminometer (Promega, Madison, WI, USA). The PB1, PA and NP genes were all from A/chicken/Shandong/l × 1023/2007 (l × 1023), while the PB2 gene was from l × 1023, belonging to the F/98-like branch and A/chicken/Shandong/196/2011(sd196), belonging to the DK1-like branch, respectively.

### 2.5. Generation of Reassortant Virus by Reverse Genetics

A recombinant rDK1-PB2-H9N2 was generated by reverse genetics. All seven internal gene segments were amplified by reverse transcription-PCR (RT-PCR) from l × 1023 and PB2 gene from DK1-like PB2 virus sd196. Each gene was individually cloned into a dual-promoter plasmid—pHW2000. To highlight that the virus is differential in the PB2 gene, we subsequently speak of l × 1023 as F/98-PB2-H9N2.

### 2.6. Library Preparation for Transcriptome Sequencing

A total amount of 1 µg RNA per sample was used as input material for the RNA sample preparations. Sequencing libraries were generated using NEBNext^®^ UltraTM RNA Library Prep Kit for Illumina^®^ (NEB, Ipswich, MA, USA) following manufacturer’s recommendations, and index codes were added to attribute sequences to each sample. 

Briefly, mRNA was purified from total RNA using poly-T oligo-attached magnetic beads. Fragmentation was carried out using divalent cations under elevated temperature in NEBNext First Strand Synthesis Reaction Buffer (5×). The first cDNA strand was synthesized using random hexamer primer and M-MuLV Reverse Transcriptase. Second strand cDNA synthesis was subsequently performed using DNA Polymerase I and RNase H. Remaining overhangs were converted into blunt ends via exonuclease/polymerase activities. After adenylation of 3′ ends of DNA fragments, the NEBNext Adaptor with hairpin loop structure was ligated to prepare for hybridization. In order to select cDNA fragments of preferentially 250~300 bp in length, the library fragments were purified using the AMPure XP system (Beckman Coulter, Beverly, CA, USA). Then, 3 µL USER Enzyme (NEB, Ipswich, MA, USA) was used with size-selected, adaptor-ligated cDNA at 37 °C for 15 min, followed by 5 min at 95 °C before PCR. Then, PCR was performed with Phusion High-Fidelity DNA polymerase, Universal PCR primers and Index (X) Primer. Finally, PCR products were purified (AMPure XP system), and library quality was assessed on the Agilent Bioanalyzer 2100 system. Overall, it is consistent with previous studies [27].

### 2.7. Quantification of Gene Expression Level and Differential Expression Analysis

FeatureCounts v1.5.0-p3 was used to count the reads numbers mapped to each gene, and then FPKM of each gene was calculated based on the length of the gene and reads count mapped to this gene. Differential expression analysis of two conditions/groups was performed using theDESeq2 R package (1.16.1). DESeq2 provide statistical routines for determining differential expression in digital gene expression data using a model based on the negative binomial distribution. The resulting *p*-values were adjusted using Benjamini and Hochberg’s approach for controlling the false discovery rate. Genes with an adjusted *p*-value < 0.05 found by DESeq2 were assigned as differentially expressed.

### 2.8. Quantitative Real-Time PCR (qRT–PCR)

Total RNA from virus-infected cells was extracted using an RNA isolation kit (Thermo Scientific, Waltham, MA, USA). First-strand complementary DNA was synthesized from 1 μg of total RNA using a TransScript RT reagent kit (TransGen, Beijing, China), and oligo dT were used for detecting host genes. Generated cDNA was subjected to qPCR in a 25 μL reaction volume using FastStart Universal SYBR Green master mix (Roche, Shanghai, China). Human *GAPDH* genes were amplified for normalization of the cDNA amount used in qPCR. Reactions were conducted in triplicate, and the data were analyzed using the 2^−ΔΔCt^ method.

### 2.9. RNA Interference

siRNAs against *SphK1* were transfected using Lipofectamine RNAiMax (Invitrogen, Carlsbad, CA, USA) at a final concentration of 20 nM following the manufacturer’s instructions. siRNAs against *SphK1* (siRNA1: 5′-GCAGCUUCCUUGAACCAUUTT-3′, siRNA2: 5′-GAUUGCUGAUGUGGACCUTAA-3′, siRNA3: 5′-GUGCACCCAAACUACUUCUTT-3′) are constructed by GenePharma Corporation (Shanghai, China).

### 2.10. Antibodies

The following antibodies were used for co-immunoprecipitation (co-IP) and Western blotting: anti-SphK1 (D1H1L, CST, Shanghai, China), anti-Actin (A1978, Sigma, Shanghai, China), anti-GAPDH (ab8245, Abcam, Shanghai, China), anti-Flag (F1804, Sigma, Shanghai, China), anti-HA (sc-805, Santa Cruz, Texas, USA), anti-influenza A virus NP (A01506, GenScript, Piscataway, NJ, USA), Mouse monoclonal anti-influenza A virus M1 antibody (Lab Homemade, Beijing, China, ZL201910912806.0).

### 2.11. Immunoprecipitation and Western Blotting

Cells were lysed in lysis buffer (50 mM Tris-Cl at pH 8.0, 150 mM NaCl, 1% Triton X-100, 1 mM DTT, 1× complete protease inhibitor cocktail, and 10% glycerol) and pre-cleared with protein G Sepharose beads (GE Healthcare, Piscataway, NJ, USA) for 2 h at 4 °C. The lysates were then immunoprecipitated with indicated antibodies or isotype-matched control antibodies plus protein G Sepharose beads at 4 °C for 2–4 h. The beads were then washed three times and boiled. Protein samples were analyzed by Western blotting. For Western blotting, protein samples were mixed with 6× loading buffer supplemented with 10% β-mercaptoethanol, heated at 100 °C for 5 min and separated on a 10% SDS–PAGE under reducing conditions. After electrophoresis, protein samples were electroblotted onto polyvinylidene difluoride membranes (Bio-Rad, Hercules, CA, USA) and blocked for 2 h in Tris-buffered saline (10 mM Tris-HCl, pH 8.0, containing 150 mM NaCl) containing 5% (*w*/*v*) non-fat dry milk and 0.5‰ (*v*/*v*) Tween-20. The blots were incubated with the primary antibodies overnight at 4 °C. The next day, the blots were incubated with corresponding horseradish peroxidase (HRP)-conjugated secondary antibodies for 1 h at room temperature (RT). HRP antibody binding was detected using a standard enhanced chemiluminescence (ECL) kit (Thermo Scientific, Waltham, MA, USA).

### 2.12. Mouse Challenge Study

A total of 33 mice (6-week-old female BALB/c mice; Vital River Laboratory, Beijing, China) were anesthetized with tiletamine-zolazepam (Zoletil; Virbac SA, Carros, France) (20 mg/g), and each mouse was inoculated intranasally with 10^6^ TCID_50_ of the indicated test virus diluted to 50 μL with phosphate-buffered saline (PBS). 5 mice from each group were monitored daily for 14 days, and mice that lost 25% of their original body weight were humanely euthanized. 9 mice from each group were used to test the pathogenicity of both viruses, and 3 mice from each group were euthanized at 3-, 5- and 7-days post-infection (dpi), respectively, for the determination of virus titers and histopathology. Nasal turbinates and lungs were collected and homogenized in 1 mL of cold PBS. Virus titers were determined by TCID_50_ assays. TCID_50_ was determined in MDCK cells with 10-fold serially diluted viruses inoculated at 37 °C for 48 h. The TCID_50_ value was calculated by the Reed–Muench method; the virus titer detection limit is 10^0.75^ TCID_50_/mL. A portion of the lung from each euthanized mouse at 5 dpi was fixed in 10% phosphate-buffered formalin, embedded in paraffin, sectioned and stained with hematoxylin and eosin (H&E), which was performed as described previously [28].

### 2.13. Statistical Analysis

Experimental groups were statistically compared by ANOVA. *p* < 0.05 was considered to indicate a statistically significant difference.

## 3. Results

### 3.1. DK1-like PB2 Enhanced the Replication Ability and Polymerase Activity of H9N2 AIV in Mammalian Cells

In this study, the evolution of the PB2 gene of H9N2 AIV in China was demonstrated, and the phylogenetic tree was then constructed using the PB2 gene sequences of all sources of H9N2 AIV from Mainland China and Hong Kong, China from 1994 to 2020. We found that the DK1-like PB2 gene is still the predominant lineage in China (Figure S1A,B), and contributes to H9N2 AIV infections in humans (Figure S1A).

**Figure 1:**
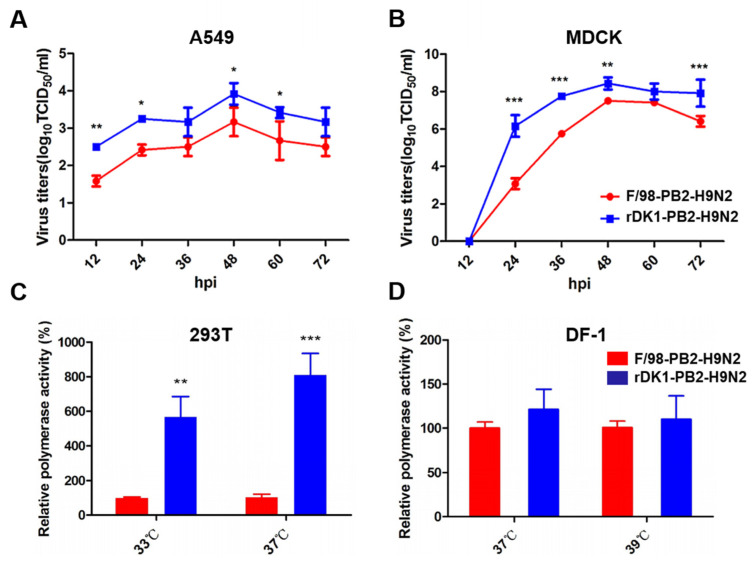
DK1-like PB2 enhances the replication ability and polymerase activity of H9N2 viruses in mammalian cells. Two strains of rDK1-PB2-H9N2 and F/98-PB2-H9N2 infected (**A**) A549 cells and (**B**) MDCK cells with MOI = 0.1, respectively, and cell supernatants were collected at 12, 24, 36, 48, 60, and 72 h post-infection to determine viral titers using the TCID_50_ assays; PB1, PA and NP proteins were co-transfected with DK1-like PB2 protein or F/98-like PB2 protein into (**C**) 293T or (**D**) DF-1 cells, respectively. After transfection, the polymerase activity was detected after culturing at different temperatures for 24 h. The polymerase activity of the F/98-like PB2 protein group was set as 100%, and the polymerase activity value of the DK1-like PB2 protein group was a percentage relative to the polymerase activity value of the F/98-like PB2 protein group. Shown in the figure are the mean ± SD of the results of 3 experiments. Differences were considered statistically significant at *p* < 0.05 (* *p* < 0.05, ** *p* < 0.01, and *** *p* < 0.001, ANOVA).

To study the effect of the DK1-like PB2 gene on the adaptability of H9N2 virus in mammalian cells, we used an H9N2 virus with the F/98-like PB2 gene as the backbone and rescued a recombinant virus named rDK1-PB2-H9N2; this was achieved by replacing the PB2 gene with a DK1-like gene using reverse genetic technology. In addition, we used a backbone virus named F/98 -PB2-H9N2 as a control. We found that replacing the DK1-like PB2 gene significantly enhanced the ability of H9N2 virus to replicate in A549 and MDCK cells (Figure 1A,B). Consistent with this, and compared with the F/98-like PB2 gene, the DK1-like PB2 gene significantly increased the polymerase activity of H9N2 AIV on human 293T cells at 33 °C (the temperature of the human upper respiratory tract) and 37 °C (the temperature of the human lower respiratory tract) (Figure 1C) but had little effect on the polymerase activity of H9N2 virus on avian DF-1 cells (Figure 1D). This suggests that the DK1-like PB2 gene enhances the replication ability and polymerase activity of H9N2 AIV in mammalian cells but has a limited effect on its polymerase activity in avian cells.

### 3.2. DK1-like PB2 Enhanced the Replication Ability and Pathogenicity of H9N2 AIV in Mice

**Figure 2:**
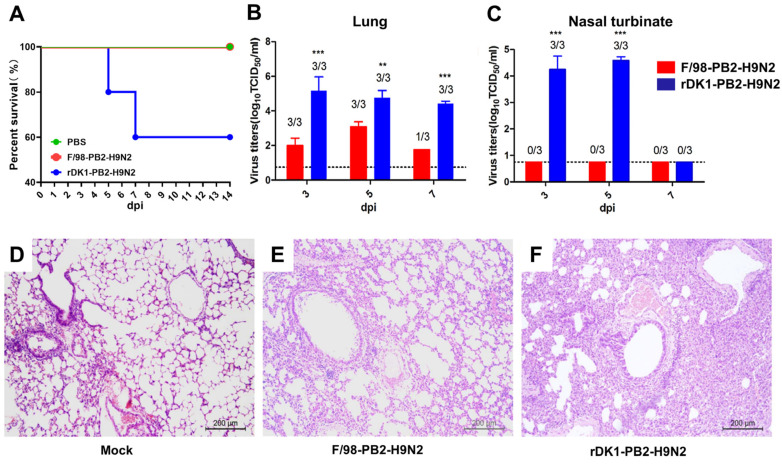
DK1-like PB2 enhances the replication and pathogenicity of H9N2 AIV in mice. Mice were infected with the virus at 10^6^ TCID_50_, the survival of the mice was monitored within (**A**) 14 days and the (**B**) lungs and (**C**) turbinates of the mice were taken 3, 5 and 7 days after infection to detect the virus titer using the TCID_50_ assays; (**D**–**F**) The lung samples infected with the virus for 5 days were fixed with 10% phosphate-buffered formalin, paraffin sections were made, and HE staining was performed to observe the pathological damage of the lungs of mice. Differences were considered statistically significant at *p* < 0.05 (** *p* < 0.01, and *** *p* < 0.001, ANOVA).

To further evaluate the effect of the DK1-like PB2 gene on the replication ability and pathogenicity of H9N2 virus in mammals, we infected mice with the virus at 10^6^ TCID_50_ and found that the H9N2 virus derived from the DK1-like PB2 gene could cause the mice to die. The survival rate was 60% (3/5), while all mice in the F/98-PB2-H9N2 group survived (Figure 2A). Furthermore, virus titers were measured by collecting lungs and nasal turbinates from the mice on days 3, 5 and 7 after virus infection, and the results showed that the lung virus titers of rDK1-PB2-H9N2 virus-infected mice were significantly higher (*p* < 0.01) than those of F/98-PB2-H9N2 virus-infected mice, with 100-fold and 10-fold higher lung virus titers than those of F/98-PB2-H9N2-infected mice on postinfection days 3 and 5, respectively. High virus titers were still detectable in the lungs of all three mice infected with rDK1-PB2-H9N2 virus on day 7 after virus infection, but only one of the mice infected with F/98-PB2-H9N2 virus could detect the virus (Figure 2B). In addition, the rDK1-PB2-H9N2 virus replicated in the nasal turbinates of mice, with viral titers reaching 10^4.25^ TCID_50_/mL (day 3) and 10^4.58^ TCID_50_/mL (day 5), but no virus was detected in the nasal turbinates of F/98-PB2-H9N2 virus-infected mice (Figure 2C). Finally, we collected lung tissues from mice 5 days postinfection for fixation and HE staining to observe histopathological changes. Compared with the control group, F/98-PB2-H9N2 infection led to mild interstitial pneumonia in mice, and only slight alveolar wall thickening, and a small amount of inflammatory cell infiltration was observed (Figure 2D,E). However, viral infection with rDK1-PB2-H9N2 resulted in more severe lesions in the lungs of mice, as evidenced by severe interstitial pneumonia, in which pulmonary hemorrhage, edema, severe alveolar wall thickening and massive inflammatory cell infiltration were observed (Figure 2D,F). In conclusion, the DK1-like PB2 gene enhanced replication of the H9N2 virus in mice and enhanced its pathogenicity in mice.

### 3.3. Activation of Host SphK1 Expression during Infection with DK1-like PB2 Gene H9N2 AIV

**Figure 3:**
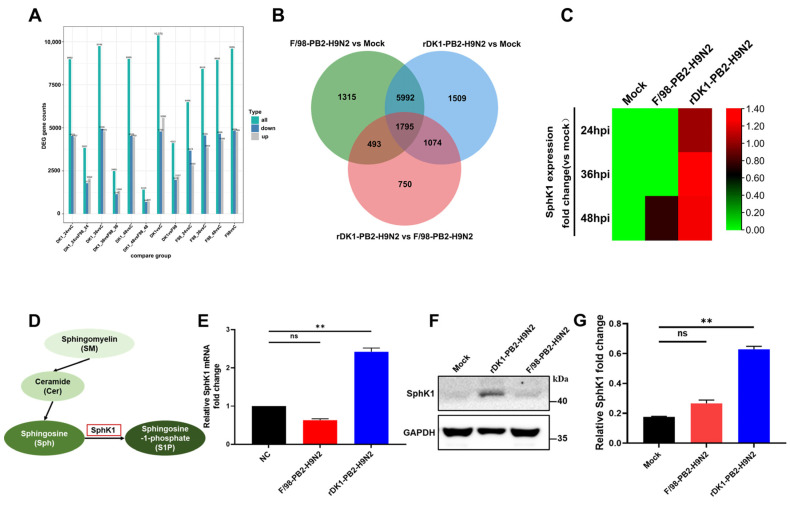
Transcriptomic screening for key differentially expressed host *SphK1* gene. (**A**) The number of differential genes in each group was obtained by transcriptomic detection. Blue and gray represent up-regulated and down-regulated differential genes, respectively, and the numbers on the columns represent the number of differential genes; (**B**) Venn diagram of the number of differential genes compared between different groups, with different colors indicating different combinations of comparisons; (**C**) fold change value of *SphK1* gene after influenza virus infection compared with the Mock group, presented as a heat map, the darker the red color, the larger the value; (**D**) schematic diagram of the role of SphK1 in cellular metabolism; (**E**) A549 cells were infected with the virus at MOI = 0.2, Cells were collected 24 h after infection to detect the mRNA expression of SphK1; (**F**) A549 cells were infected with the virus at MOI = 0.2, and the cells were collected 24 h after infection to detect the expression of SphK1 protein, glyceraldehyde-3-phosphate dehydrogenase (GAPDH) was used as a loading control. (**G**) for (**F**) densitometry analysis quantification. Differences were considered statistically significant at *p* < 0.05 (** *p* < 0.01, ANOVA).

We demonstrated that H9N2 viruses with different lineages of PB2 genes have different mammalian adaptations, and we sought to find differentially expressed host factors following infection by these two viruses. To screen for differential host genes after infection with H9N2 viruses with different lineages of PB2 genes, we infected A549 cells using F/98-PB2-H9N2 and rDK1-PB2-H9N2 and collected cell samples 24, 36, and 48 h after infection for transcriptomic assays. A total of 4112 common differentially expressed genes were found at three time points after viral infection in both groups (Figure 3A,B and Figure S2). Among them, we focused on *SphK1* because its expression was upregulated at all three time points of rDK1-PB2-H9N2 infection, while its expression was basically unchanged during F/98-PB2-H9N2 infection (Figure 3C). SphK1 is a key kinase in the sphingomyelin metabolic pathway (Figure 3D) [21], and we confirmed that the mRNA expression of SphK1 was upregulated during rDK1-PB2-H9N2 infection by quantitative real-time PCR (qRT–PCR) (Figure 3E). This phenomenon was further confirmed by western blotting (Figure 3F,G). The expression of SphK1 was upregulated during rDK1-PB2-H9N2 infection but not during F/98-PB2-H9N2 infection. As an immune regulator of the host, we wondered whether the upregulation of SphK1 during rDK1-PB2-H9N2 infection is the basis of its mammalian adaptation or is a defense mechanism activated by the host to defend against a strongly virulent strain, and we tried to answer this key question.

### 3.4. SphK1 Inhibits the Replication of DK1-like PB2 Gene H9N2 AIV In Vitro

**Figure 4:**
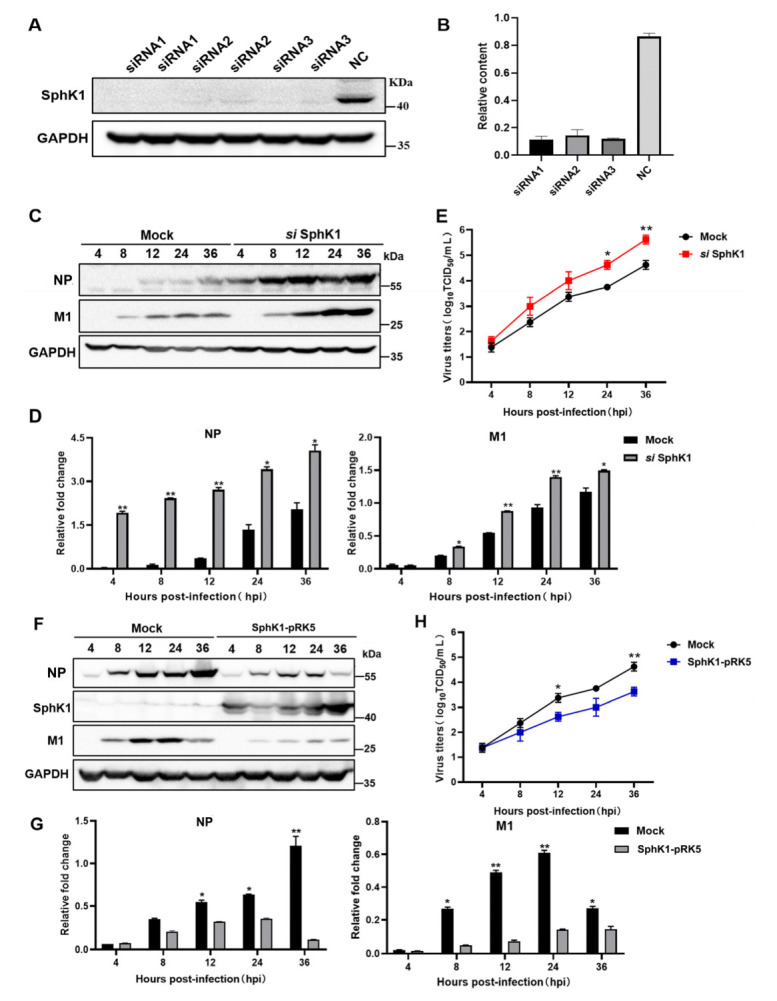
SphK1 protein inhibits H9N2 virus protein expression and virus replication in A549 cells. (**A**) Three designed siRNAs targeting *SphK1* and negative control (NC) were transfected into A549 cells, and cells were collected 24 h after transfection for western blotting (WB) detection and (**B**) densitometry analysis and quantification; (**C**) siRNA 1 and NC was pretransfected into A549 cells, the virus was then infected with the virus at MOI = 0.5, and the cells were collected for WB at 4, 8, 12, 24 and 36 h after infection, and the expression levels of viral NP, M1 and GAPDH proteins were detected and performed (**D**) densitometry analysis Quantification; (**E**) siRNA 1 was pretransfected into A549 cells, and then the cells were infected with the virus at MOI = 0.5. Cell supernatants were collected at 4, 8, 12, 24 and 36 h after infection for TCID_50_ assay to determine virus titer. (**F**) The SphK1-pRK5 (SphK1-Flag-pRK5) and Flag-pRK5 empty vector was pretransfected into A549 cells, then the cells were infected with the virus at MOI = 0.5, and the cells were collected for WB at 4, 8, 12, 24 and 36 h after infection. Detect the expression levels of SphK1, viral NP, M1 and GAPDH proteins and perform (**G**) densitometry analysis and quantification; (**H**) The constructed SphK1 eukaryotic expression vector was pretransfected into A549 cells, and then the cells were infected with the virus at MOI = 0.5. Cell supernatants were collected at 4, 8, 12, 24 and 36 h after infection for TCID_50_ assay to determine virus titer. GAPDH was used as a loading control. Shown in the figure is the mean ± SD of the results of 3 trials. Differences were considered statistically significant at *p* < 0.05 (* *p* < 0.05, ** *p* < 0.01, ANOVA).

To clarify the role of SphK1 protein during the infection of the DK1-like PB2 gene H9N2 virus, three siRNAs of SphK1 were designed, and eukaryotic overexpression plasmids of SphK1 protein were constructed in this study. First, the interference effect of siRNA on SphK1 was verified. The interference effect of all three siRNAs was acceptable, and the first siRNA was selected for subsequent experiments (Figure 4A,B). Subsequently, the rDK1-PB2-H9N2 virus was used to infect A549 cells with SphK1 protein knockdown, and the results showed that the expression of viral NP and M1 proteins was significantly increased (Figure 4C,D). Additionally, the viral replication titer was significantly increased at 24 hpi (*p* = 0.0198) and 36 hpi (*p* = 0.0089) (Figure 4E). At the same time, by infecting A549 cells with SphK1 protein overexpression, it was found that overexpression of the SphK1 protein significantly inhibited the expression of viral NP and M1 proteins (Figure 4F,G and Figure S3), and consistent with this finding, rDK1-PB2-H9N2 viral replication titers also decreased at 12 (*p* = 0.0354) and 36 hpi (*p* = 0.0078) (Figure 4H). The above results suggest that the SphK1 protein has an inhibitory effect during the infection of the DK1-like PB2 gene H9N2 virus, and this effect is a defense mechanism activated by the host to resist the infection of the H9N2 virus with strong replication ability. Why does the mammalian adaptation of the DK1-like PB2 gene H9N2 virus remain stronger than that of the F/98-like gene H9N2 virus despite the upregulation of SphK1 expression by the host during infection with the DK1-like PB2 gene H9N2 virus? This led us to speculate whether there is a mechanism for the DK1-like PB2 gene H9N2 virus to escape the inhibitory effect of SphK1.

### 3.5. The Interaction between SphK1 Protein and DK1-like PB2 Protein Is Weaker Than That with F/98-like PB2 Protein

**Figure 5:**
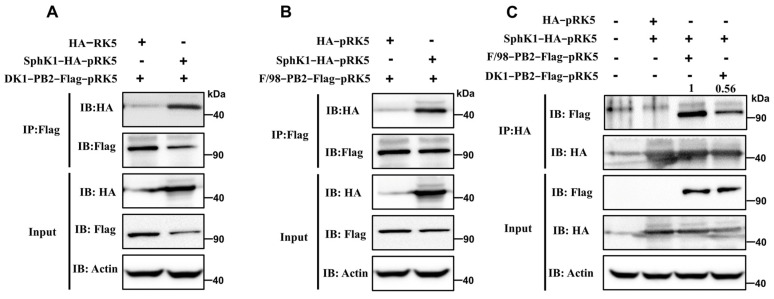
Interaction of SphK1 with PB2 proteins of different lineages. According to the plasmid transfection mode shown in the figure, 2 μg of the corresponding plasmids were transfected in the corresponding 293T cell wells. The cells were collected 24 h after transfection, immunoprecipitated with Flag antibody, and Western blotting was performed with HA and Flag antibody to detect whether (**A**) the DK1-like PB2 protein and (**B**) the F/98-like PB2 protein can interact with the SphK1 protein; (**C**) According to the plasmid transfection mode shown in the figure, 2 μg of the corresponding plasmids were transfected in the corresponding 293T cell wells. The cells were collected 24 h after transfection, and HA antibody was used for immunoprecipitation, and HA and Flag antibodies were used for Western blotting test to detect the interaction strength of SphK1 protein with F/98-like PB2 protein and DK1-like PB2 protein (IP: immunoprecipitation, IB: immunoblotting, Input: refer to whole cell lysates, Actin was used as a loading control).

To further investigate whether the DK1-like PB2 gene H9N2 virus has a mechanism to escape the inhibitory effect of host SphK1, we examined the interactions of SphK1 protein with DK1-like PB2 protein and F/98-like PB2 protein. The results showed that the SphK1 protein can interact with both the DK1-like PB2 protein and the F/98-like PB2 protein (Figure 5A,B), but the interaction between the SphK1 protein and the DK1-like PB2 protein is weaker than that of F/98-like PB2 protein (Figure 5C). The PB2 protein is a key component protein of the influenza virus polymerase complex, and the binding of SphK1 protein to the PB2 protein likely hinders the assembly of the virus polymerase complex, leading to a decrease in viral polymerase activity (Figure 1C), thereby inhibiting viral replication.

## 4. Discussion

H9N2 AIV has been circulating in China for more than 20 years and has undergone a series of gene reassortments and mutations [4,6,21,22]. The PB2 gene is a key gene for virus replication and cross-species transmission and its mutation and reassortment are particularly frequent. PB2 exhibits genetic diversity due to a series of reassortments, which resulted in the predominance of different lineages of PB2 genes at different periods in China, including the dominant BJ/94-like lineage in 1994, the subsequent G1-like and F/98-like lineage and the current DK1-like lineage in the H9N2 virus. The replacement of the previously prevalent F/98-like PB2 gene by the DK1-like PB2 gene in H9N2 virus has evolutionary importance. In general, the mammalian adaptation of AIV depends on the gradual accumulation of adaptive mutations during the replication and transmission of the virus in mammals [29,30,31]. However, the DK1-like PB2 gene was directly reassorted from the waterfowl virus into the chicken H9N2 virus, and we found that compared with the F/98-like PB2 gene H9N2 AIV, the DK1-like PB2 gene enhanced the replication ability and polymerase activity of H9N2 virus in mammalian cells in vitro and increased its replication ability and pathogenicity in mice. These results indicate that the DK1-like PB2 gene H9N2 virus was well adapted to mammals without undergoing adaptation in mammals and deserves to be further investigated.

In the study, we found that compared with the F/98-like PB2 gene H9N2 AIV, the Dk1-like PB2 gene enhanced the replication ability and polymerase activity of H9N2 virus in mammalian cells in vitro and increased its replication ability and pathogenicity in mice. Through transcriptomic data analysis and experimental verification, we screened the *SphK1* gene. SphK1 is a key enzyme that catalyzes the production of sphingosine-1-phosphate from ceramide and sphingosine and is involved in the regulation of cell proliferation and apoptosis, vasoconstriction and remodeling, inflammation and metabolism, and other physiological functions and is an important immune regulator [21,22,32]. 

Previous studies have shown that the inhibition of sphingosine kinase by bovine viral diarrhea virus NS3 is crucial for efficient viral replication and cytopathogenesis [33], and that the SphK1 activator K6PC-5 can alleviate Ebola virus infections [34]. However, Seo et al. found that SphK1 plays a positive role in WSN (H1N1) virus infection and can promote the susceptibility of cells to the virus [35,36]. This suggests that the SphK1 protein may play different roles in different viral infections, or it may have multiple roles, and its role varies with the pathogenicity of the virus. In our study, we found that ShpK1 was upregulated during infection with the more mammalian-adapted DK1-like PB2 gene H9N2 AIV, while there was no significant change during infection with the F/98-like PB2 gene H9N2 AIV, and virus replication was inhibited by the SphK1. Co-IP assays further showed that the SphK1 protein had a weaker ability to bind the DK1-like PB2 protein than the F/98-like PB2 protein, which may be the molecular basis by which the DK1-like PB2 gene H9N2 AIV escapes the inhibitory effect of the immune factor SphK1. However, the specific pathway and mechanism for the role of SphK1 protein during H9N2 AIV infection remain to be further investigated.

In conclusion, the struggle between the virus and host is endless. The DK1-like PB2 gene was introduced into H9N2 AIV during viral evolution, making the virus more adaptable in mammals. As a multifunctional protein, PB2 may antagonize host defense through other unknown mechanisms. Understanding these mechanisms will help us to better assess the cross-species risk of the viruses and discover new antiviral drugs. In addition, it is worth mentioning that a recently isolated swine influenza virus reassorted with H9N2 in Hong Kong shows significant risk of becoming a human pandemic [37]. Therefore, continued attention should be paid to the H9N2 virus and its reassortants in animals and humans.

## Data Availability

Data is contained within the article.

## Supplementary Materials

The following supporting information can be downloaded at: https://www.mdpi.com/article/10.3390/v14071585/s1, Figure S1: The DK1-like PB2 gene is still the dominant lineage in prevalent H9N2 AIV in China; Figure S2: Differential gene clustering after H9N2 virus infection of A549 cells; Figure S3: Overexpression of the SphK1 protein inhibits the protein expression of H9N2 virus in A549 cells; Table S1. Sequence information used in this study.
