# Supplementary material for: Effect of the Interaction between Viral PB2 and Host SphK1 on H9N2 AIV Replication in Mammals
Source: Viruses. 2022 Jul 21;14(7):1585. doi: 10.3390/v14071585 (PMC9322132; doi:10.3390/v14071585)
Supplement: Supplementary file 1 [file viruses-14-01585-s001.zip › Supplementary files/Figures S1-S3.pdf]

Supplementary material

Effect of the Interaction Between Viral PB2 and Host SphK1 on H9N2 AIV Replication in Mammals

Yong Zhou [1,†], Weihua Gao [1,†], Yan Sun [1], Yuxin Guo [1], Juan Pu [1,*] and Yuping Wu [2,*]

[1] Key Laboratory for Prevention and Control of Avian Influenza and Other Major Poultry Diseases, Ministry of Agriculture and Rural Affairs, College of Veterinary Medicine, China Agricultural University, Beijing, 100193, China; zhouyong@cau.edu.cn (Y.Z.); gwh212@163.com (W.H.-G.); yyyyyan111@163.com (Y.S.); yuuux-in_guo@163.com (Y.X.-G.); pujuan@cau.edu.cn (J.P.)

[2] College of Life Science and Basic Medicine/Center for Biotechnology Research, Xinxiang University, Xinxiang 453003, China; wuyuping62@xxu.edu.cn (Y.P.-W)

† These authors contributed equally to this work.

* Correspondence: wuyuping62@xxu.edu.cn (Y.P.-W); Tel.: +86 13663739996 ; pujuan@cau.edu.cn (J.P.); Tel.: +86 13810129404

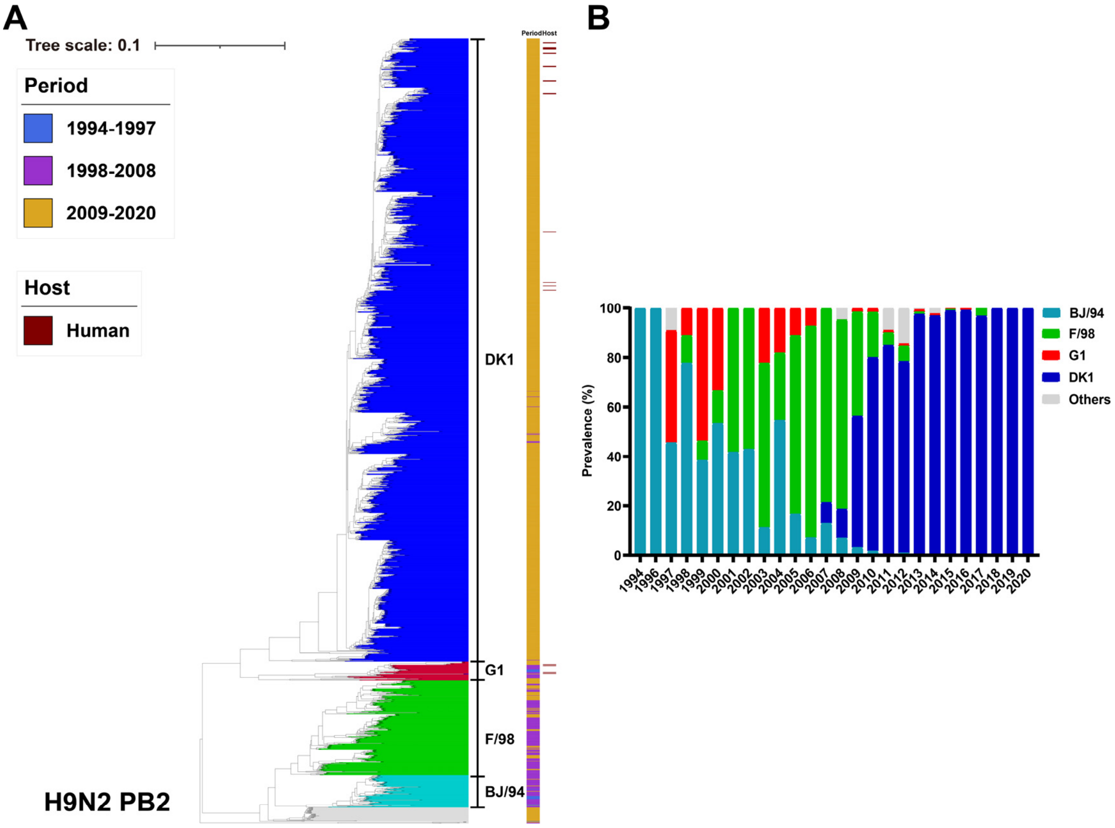

Figure S1. The DK1-like PB2 gene is still the dominant lineage in prevalent H9N2 AIV in China. (A) The H9N2 virus PB2 gene sequences from 1994-1998 in China were used to construct a phylogenetic tree using IQtree software based on the maximum likelihood method, and the BJ/94-like, F/98-like, G1-like and DK1-like lineage PB2 strains were shown in cyan, green, red and blue, respectively, on the evolutionary tree. iTOL was used to annotate the strains with different periods and hosts. (B) Statistics on the dynamic prevalence of different lineages of the PB2 gene of H9N2 virus in China for each year during 1994-2020.

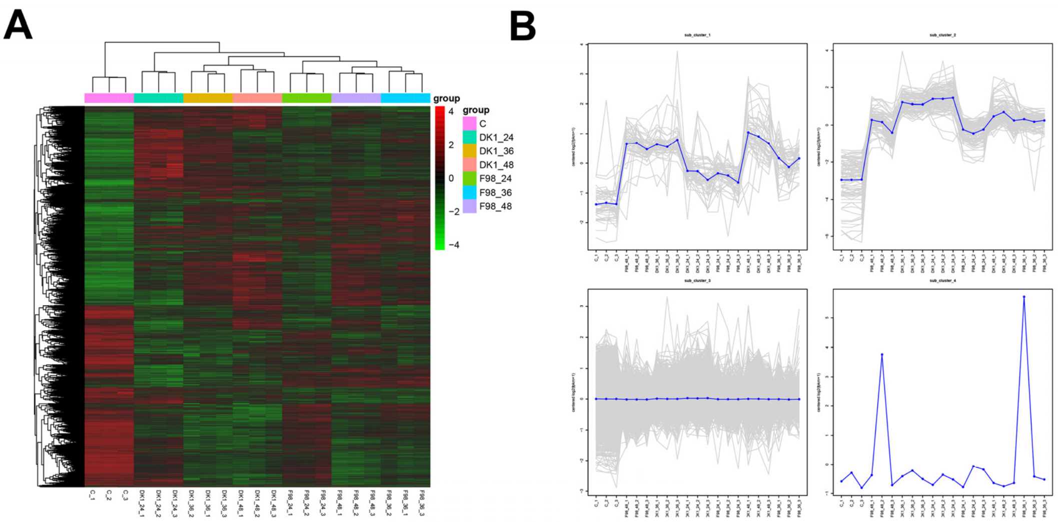

Figure S2. Differential gene clustering after the viral infection of H9N2 in A549 cells. (A) Heatmap of differential gene clustering between different groups (union of differential genes compared with all combinations); (B) line chart of differential gene clustering between different groups, clustered by taking log2(fpkm+1) for differential gene expression with the H-cluster method and centering correction. The differentially expressed genes were divided into 4 clusters, and the genes in the same cluster had similar expression trends under different treatment conditions. The abscissa is the sample name, and the ordinate is the expression value after taking the logarithm and correcting for centering. The gray line in each subplot represents the relative corrected gene expression level of genes in a cluster under different experimental conditions, and the blue line indicates the average of the relative corrected gene expression level of all genes in this cluster under different experimental conditions.

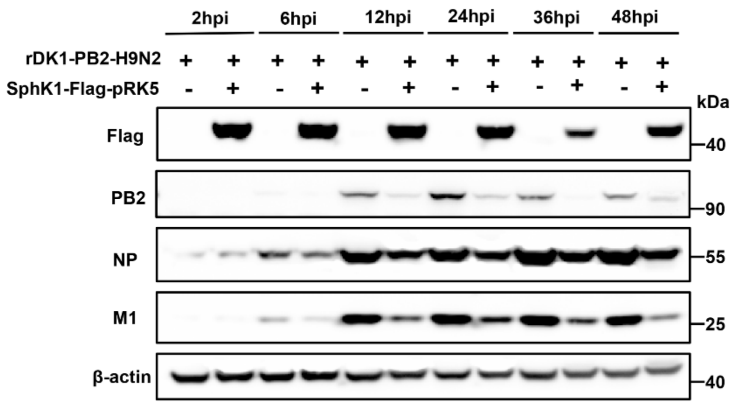

Figure S3. Overexpression of the SphK1 protein inhibits the protein expression of H9N2 virus in A549 cells. The SphK1-Flag-pRK5 and Flag-pRK5 empty vector was pretransfected into A549 cells, after which the cells were infected with virus at MOI=0.5, and the cells were collected for WB at 2, 6, 12, 24, 36, and 48 hours postinfection to detect the expression of viral PB2, NP, and M1 proteins, β-actin was used as a loading control.
